# Operative Management of Sciatic Nerve Palsy due to Impingement on the Metal Cage after Total Hip Revision: Case Report

**DOI:** 10.1155/2011/830296

**Published:** 2011-07-11

**Authors:** Alessandro Bistolfi, Giuseppe Massazza, Davide Deledda, Elisa Lioce, Maurizio Crova

**Affiliations:** ^1^Department of Orthopedics, Traumatology, and HM, CTO/M Adelaide Hospital, Turin, Italy; ^2^University of the Studies of Turin, Turin, Italy

## Abstract

This paper discusses a sciatic nerve palsy developed after a right total hip revision with a Burch-Schneider metal cage. A sciatalgic nerve pain appeared after surgery, while the palsy developed in about fifteen days. An electromyography showed the delay of the nerve impulse gluteal level. During the surgical exploration of the hip, a compression of the nerve on the metal cage was observed. The nerve was isolated, released from the fibrotic tissue and from the impingement, and was protected with a muscular flap. The recover from the pain was immediate, while the palsy recovered one month later.

## 1. Introduction

Sciatic nerve palsy is a known complication following total hip arthroplasty (THA) [1–3] and revision [4], with an incidence from 0.2 to 2.8% and from 1.6% to 7.6%, respectively; it may result from a direct [5, 6] or from an indirect injury [7], while sometimes, it can be idiopathic [2]. The anatomical variants of the nerve course [8] (e.g., the passage of the sciatic nerve through the piriformis muscle) and the transtrochanteric and the posterior approaches [3] can be adjunctive causes of risk. In complex reconstructive revisions, the lesion of the nerve in association with the acetabular cages and rings was reported [9, 10]. 

We report a case of sciatic nerve palsy following THA revision due to compression and impingement on the lateral edge of the metallic acetabular reinforcement ring without course nerve alterations. With this paper we want to demonstrate that the palsy could be completely and immediately recovered with the positioning of a muscular flap between the nerve and the edge of the metal ring associated with a revision debridement surgery.

## 2. Case Presentation

**Figure 1:**
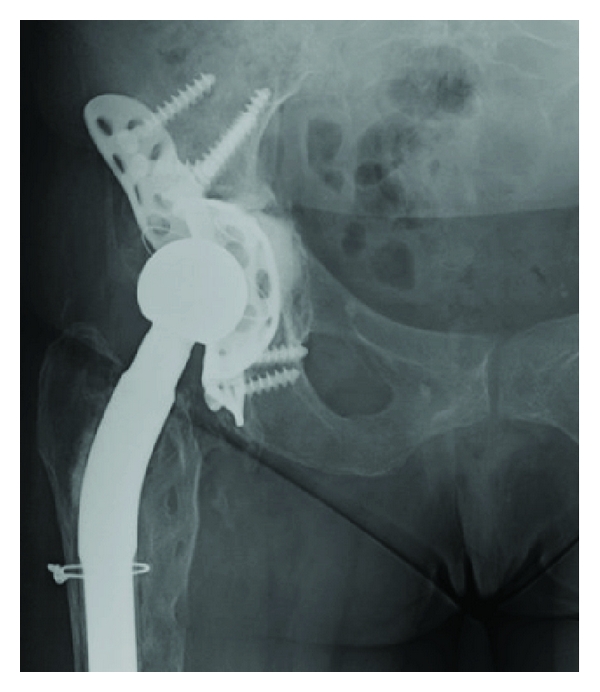
Radiological anteroposterior radiographs of the pelvis showing the Burch Schneider metal cage.

**Figure 2:**
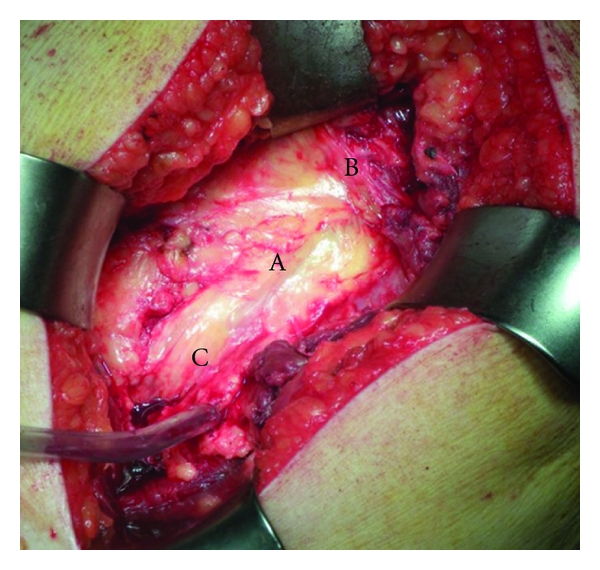
The sciatic nerve (A) compressed by fibrotic tissue (B) after the section of the external rotator muscles (C).

**Figure 3:**
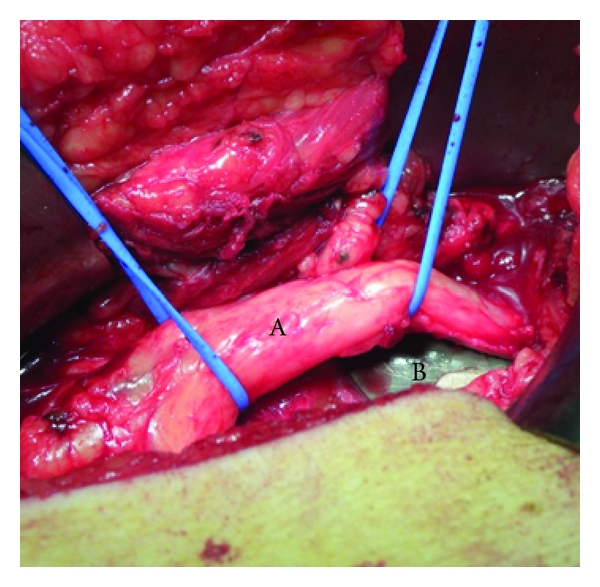
The sciatic nerve (A) impinging on the metal cage (B).

**Figure 4:**
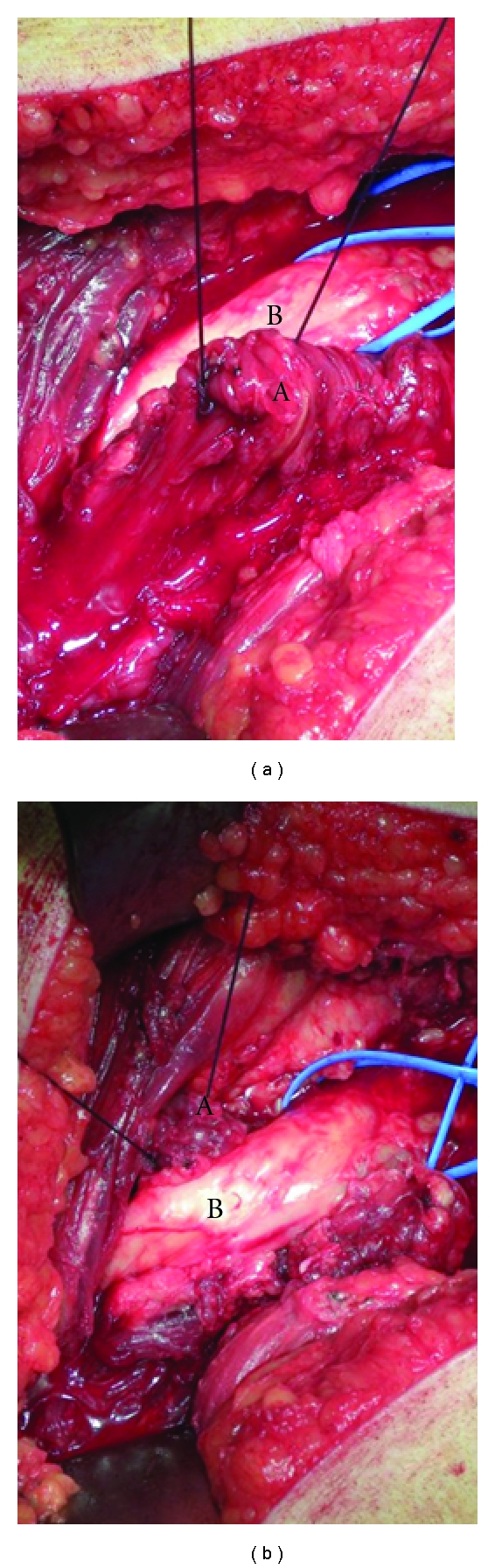
The muscular flap (A) positioned between the sciatic nerve (B) and the metallic cage: (a) preparation of the flap, (b) the flap in its final position.

A right total hip revision was performed in a 56-year-old woman for aseptic loosening of the primary THA. The primary arthroplasty was implanted 14 years before that for rheumatoid arthritis. During the revision surgery, a Burch-Schneider metal cage was used to treat the acetabular bone defect. During the first postoperative week, a typical right (homolateral) sciatalgic pain developed in association with a severe lumbar back pain. The position with the knee flexed of approximately 30° provided moderate pain relief. The Tinel, Lasegue, and Valleix tests were all positive on the right side. After six days of pharmacological treatment, the sciatalgic pain was evaluated with a magnetic resonance (MR) which demonstrated L1-L2 and L3-L4 herniated discs, but no specific radicular compression.The radiological evaluation of the prosthesis did not show mobilization or malpositioning of the implant (Figure 1). After approximately ten days, a palsy of moderate grade of the extensor hallucis longus (EHL) and of the extensor digitorum communis (EDC) arose in association with a low-grade hypoesthesia of the posterior plantar region of the right foot. The electromyograph test performed after the pharmacological treatment showed a high-grade delay of the nerve impulse at gluteal level. The hypothesis was of a compression of the sciatic nerve directly on the cage by a fibrotic scar tissue. Therefore, revision surgery and debridement of the sciatic nerve at the hip were planned. The posterior approach, following the previous exposure, was used. After the section of the tendon of the external rotator muscles group and of the piriformis muscle, the nerve appeared clearly embedded in a fibrotic tissue (Figure 2). Moreover, a straight relationship between the sciatic nerve and the Schneider ring was found (Figure 3): this relationship caused the compression of the nerve itself on the metallic cage, particularly during the movement of flexion of the hip, which clearly stretched the nerve. No anatomical alterations of the sciatic nerve through the piriformis muscle were observed. The Schneider cage appeared well positioned, so it was not necessary to revise the ring. The nerve was carefully released from the fibrotic tissue, and a muscular flap [11] was made using part of the dissected profundus gluteus muscle to allow a nerve glide on the ischiatic bone and on the edge of the Burch Schneider metal cage (Figure 4). During the second day after the operation, the patient had pain relief, improvement of the palsy, and the possibility to move the hip without pain (negative Tinel and Lasegue tests). After 45 days, the patient was reevaluated: the hypoesthesia and the palsies were completely cleared up.

## 3. Discussion

It is difficult to establish the precise aetiology of acute sciatic nerve palsies after revision surgery [2, 5]. To our knowledge, a conflict of the sciatic nerve (compression) on the metallic acetabular cage without anatomical variations of the sciatic nerve course is an uncommon situation. In this presented case, the pain appeared during the first week after the surgery, and the palsy developed during the rehabilitation and increased constantly. The radiographs did not show any alteration of the prosthesis, and the lumbar MR was negative for radicular compression. On the contrary, the electromyograph test was fundamental to confirm the diagnosis of nerve palsy at the level of the hip and, therefore, to decide for a revision surgery.

Through the posterior approach, the nerve was immediately isolated and released from the fibrotic tissue and from the impingement on the metal ring and was protected with a muscular flap. Due to the early onset of the symptoms, it is probable that the impingement between the nerve and the metallic ring was the primary cause of the pain. On the contrary, the fibrotic tissue probably developed after few days and contributed to the development of the neurological deficits. Without anatomical variations of the nerve course, it is difficult to anticipate in which patients the nerve palsy will develop. We think that depending on personal characteristics the nerve stretching or compression could evidence different grade of clinical symptoms. In those cases, which we define idiopathic, the only treatment is to perform a muscular flap to let the physiological nerve glide during the hip flexion. The benefits and the recovery from pain is immediate, while the complete recovery of the palsy takes few days. This case shows how the presence of acute sciatic palsies and pain after hip surgery can be reasonable to explore the hip, especially when, being excluded all the other causes of sciatalgic pain or palsy, the diagnosis is documented with a positive electromyograph test [12]. 

In conclusion, the entrapment or impingement of the sciatic nerve should be suspected when a sudden sciatalgic pain or palsy develops after THA revision and in particular when a reinforcement ring is used. The nerve should be assessed using the electromyograph. Once the diagnosis is confirmed and any other cause is excluded (anatomical variations of the nerve course, direct injuries, etc.), the revision surgery must be promptly performed using a soft muscular flap to eliminate the palsy of the sciatic nerve especially in case of impingement on the rim of the metallic cage.
